# “Biqi” Bayberry Extract Promotes Skeletal Muscle Fiber Type Remodeling by Increasing Fast Myofiber Formation via the Akt/FoxO1 Pathway in Mice

**DOI:** 10.3390/foods12132471

**Published:** 2023-06-23

**Authors:** Jinjie Li, Yi Li, Xiangying Suo, Jiangtao Li, Da Huang, Guangning Kou

**Affiliations:** 1 Centre for Nutritional Ecology and Centre for Sport Nutrition and Health, Zhengzhou University, Zhengzhou 450001, China; lijinjie@gs.zzu.edu.cn (J.L.); ljt15038542634@163.com (J.L.); huangda13103937194@163.com (D.H.); 2 Zhejiang Citrus Research Institute, Taizhou 318000, China; m_liyi@163.com; 3 Department of Nutrition and Food Hygiene, School of Public Health, Zhengzhou University, Zhengzhou 450001, China; xy_suo123@163.com

**Keywords:** bayberry extract, skeletal muscle, fast-twitch fibers, myofiber type, FoxO1

## Abstract

Bayberry is known to be a rich source of natural flavonoids and has been reported to have various health-promoting abilities. However, its function on regulating skeletal muscle fiber type remains unclear. This study examined whether bayberry extract affects skeletal muscle fiber type by promoting fast myofiber formation, as well as the potential molecular mechanism. After eight weeks, the “Biqi” bayberry extract (BBE) groups mice displayed markedly enhanced grip strength and improved metabolic rate compared to the control group mice. BBE also significantly increased myofibers size, LDH activity, MyHC-IIb (fast-twitch mRNA) expression, and the percentage of fast-twitch myofibers, while decreasing SDH activity, MyHC-I (slow-twitch mRNA) expression, and slow-twitch myofibers percentage in the skeletal muscle of the mice. The effect of BBE on regulating skeletal muscle fiber type remodeling is likely attributed to its activation of the Akt-FoxO1 pathway. Our findings indicated that BBE can effectively regulate the expression and proportion of fast-twitch fibers, making it a potential therapy for improving glucose homeostasis of skeletal muscle.

## 1. Introduction

Skeletal muscle, the largest tissue in mammals, accounts for approximately 40% of the mass of the whole body [1]. On the basis of subtype classification of myosin heavy chain (MyHC), skeletal muscle is composed of slow-twitch (red-colored, MyHC-I), fast-twitch (white-colored, MyHC-IIb), and middle-type (MyHC-IIa and MyHC-IIx) muscle fibers [2]. Slow-twitch myofibers, rich in mitochondria and myoglobin, are marked by high oxidative capacity and good endurance, and rely on oxidative metabolism for energy. In contrast, fast-twitch muscle fibers exhibit fewer mitochondria, lower endurance, and greater strength, as the energy depends mainly on glycolysis [2,3]. In addition to controlling motion, muscle loss, and myofiber types are directly linked to fatigue, obesity, diabetes, and metabolic syndrome [4,5,6]. For example, accumulating studies have indicated that increasing the slow muscle fibers in skeletal muscle is advantageous for improving endurance performance, while elevating fast muscle fibers helps to maintain glucose and energy homeostasis [5].

Ignited by a range of internal and external factors (i.e., disease, hormones, nerve-activity, sport, nutrition), skeletal muscles exhibit an exceptional degree of plasticity in their ability to convert between fiber types. A recent study has shown a normal-protein ketogenic diet combined with exercise training can enhance the skeletal muscle oxidative capacity in mice [7]. Athletes in endurance have markedly more slow-twitch muscle fibers compared with power athletes and nonathletes [8], whereas the weightlifters and sprinters have a larger proportion of fast-twitch muscle fibers. However, it is impractical for most people to carry out high-intensity exercise in the long term, and endurance or explosive training triggering the transformation of myofiber type switching may lead to sports injuries. Thus, nutritional interventions that can influence the fiber types has attracted many researchers’ attention. In vitro studies have revealed that dimer procyanidin B2 can regulate the slow myofiber types remodeling through AMPK signaling [9]. In contrast, epigallocatechin gallate can significantly reduce the formation of slow myofiber by repressing the AMPK-PGC-1α pathway [10]. Furthermore, evidence in mice has also shown that grape seed proanthocyanidin extract and *Vitis vinifera* Jingzaojing leaf and shoot extract promote myofiber type switching from fast-to-slow types via different molecular mechanisms [11,12], whereas geniposide and lauric acid exhibited a stimulating effect on glycolytic fiber formation by regulating FoxO1/PDK4 signaling and TLR4 signaling pathway [5,13]. Thus, plant-based bioactive compounds have shown great potential in skeletal muscle remodeling.

The bayberry (*Morella rubra* Sieb. et Zucc.) is a subtropical fruit with popularity in southern China and other countries in eastern Asia. In addition to its desirable taste, bayberries have also been noted for multiple biological activities such as antioxidant [14], antibacterial [15], antidiarrheal [16], and antitumor activities [17]. These bioactivities are attributed to the important phytochemicals in the fruit such as anthocyanins and flavonoid glycosides. Previous studies have reported that the bayberry extract treatment can elicit beneficial effects on insulin sensitivity, glucose uptake, and glucose metabolism in various animal models [18,19]. Additionally, cyanidin-3-glucoside, one representative anthocyanin found in berries, has been tested in clinical trials for its effects on diabetes prevention, lipid modulation, and prevention of neurodegenerative diseases [20]. Similarly, the active components in bayberry extract, such as myricetin and quercetin, have been shown to activate the AMP-activated protein kinase, which can improve glucose uptake by promoting mitochondrial function [21]. These results suggest bayberry extract shows great potential in regulating glucose metabolism and homeostasis. Taken from the growing body of evidence indicating a strong correlation between fast-twitch myofiber and the regulation of glucose metabolism and homeostasis together [13,22,23], bayberry is likely to have influence on the fast-twitch myofiber. Nevertheless, the impact of bayberry extract on the composition of skeletal myofibers is rarely reported.

Given the positive effect of bayberry extract on improving glucose homeostasis, it is reasonable to hypothesize that bayberry extract has a potential function on regulating fast-twitch myofiber composition and skeletal myofiber type. In this study, we isolated and prepared water-soluble extract from “Biqi” bayberry fruit, quantified its main active compounds through liquid chromatography-mass spectrometry, and investigated its effects on exercise performance, metabolic capacity, myofiber type composition, and possible mechanism involved in mice. This study aims to evaluate the impact of bayberry extract on skeletal myofibers and explore the underlying pathway of its action, which may provide a theoretical basis for further investigation and application of bayberry extract.

## 2. Materials and Methods

### 2.1. Chemicals and Materials

Pure standards of myricetin-3-O-α-L-rhamnoside (myricitrin, purity ≥ 98%), quercetin 3-O-α-L-rhamnopyranoside (quercitrin, purity ≥ 98%), cyanidin-3-glucoside (C3G, purity ≥ 99%), myricetin (purity ≥ 98%), quercetin (purity ≥ 99%), quercetin 3-O-β-D-galactopyranoside (hyperoside, purity ≥ 98%), and kaempferol (purity ≥ 99%) were obtained from Anpel Scientific Instrument Co., Ltd. (Shanghai, China). Methanol, ammonium formate, and formic acid used for liquid chromatography were of chromatographic grade and purchased from Aladdin Co., Ltd. (Shanghai, China). Ultrapure water was purified from a Water Purification System (Bedford, MA, USA, Millipore Milli-Q Advantage A10). Macroporous resin D101 (particle size 0.3~1.2 mm) used for separation was obtained from China National Pharmaceutical Group Chemical Reagent Co., Ltd. (Shanghai, China). All other chemicals and reagents used were of analytical grade unless otherwise noted.

### 2.2. Preparation of the Bayberry Extracts

**Figure 1:**
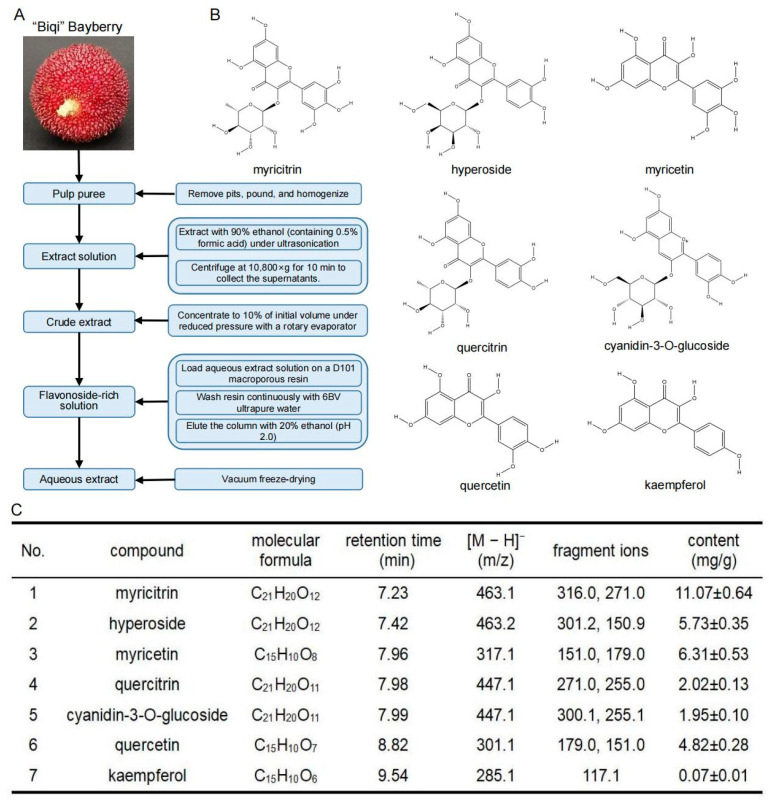
Preparation and composition analysis of “Biqi” bayberry extract. (**A**) Schematic diagram of the BBE preparation. (**B**) Structures of the predominant flavonoids in BBE: myricitrin, myricetin, hyperoside, quercetin, quercitrin, cyanidin-3-O-glucoside, kaempferol. (**C**) Determination of the main flavonoids in BBE by liquid chromatography mass spectrometry. The content of the compounds was expressed by the mean ± SD of three replicate experiments.

Bayberry fruits of the common cultivar “Biqi” were harvested at commercial maturity from Taizhou city in Zhejiang province, China. The fruits were washed, pitted, pounded, and homogenized. As depicted in Figure 1A, the extraction process was performed with 90% ethanol (containing 0.5% formic acid) under ultrasonication (180 W) at 45 °C for 50 min. Afterwards, the extract was centrifuged at 10,800× *g* for 10 min to collect the supernatants. The precipitation was then extracted twice using the above procedure. Thereafter, all supernatants combined were concentrated to 10% of initial volume under reduced pressure with a rotary evaporator (30 °C, 180 rpm, vacuum degree > 0.095 MPa) (Yarong Biochemical Instrument Factory, Shanghai, China, SHZ-III). Then, the remaining aqueous extract solution was loaded on a D101 macroporous resin column at a flow rate of 2 BV/h (where BV is a volume of liquid equal to the bed volumes). To remove soluble impurities such as sugars, the resin was continuously washed with 6 BV ultrapure water (3 BV/h). Then, the column was eluted with 20% ethanol (pH was adjusted to 2.0 with hydrochloric acid) to obtain the anthocyanin- and flavonoside-rich parts. After performing a rotary evaporation for ethanol removing and further concentration (30 °C, 180 rpm), the extracts were vacuum-dried with a freeze dryer (Songyuanhuaxing Technology Co., Ltd., Beijing, China, LGJ-10FD) to obtain the “Biqi” bayberry extract (BBE) for further analysis.

### 2.3. Quantification of Main Flavonoid Profiles by LC-MS/MS

Phytochemical quantification of the extracts was conducted using a hybrid triple-quadrupole mass spectrometer coupled with a liquid chromatography system (Applied Biosystems, Foster, CA, USA, 5500+ QTRAP). Chromatographic separation was achieved using a C18 analytical column (100 × 4.6 mm, 5 μm). The column was kept at 25 °C for the chromatographic separation with mobile phase A (2 mM ammonium formate aqueous solution containing 0.01% formic acid) and mobile phase B (2 mM ammonium formate in methanol with 0.01% formic acid) at a flow rate of 0.4 mL/min. The gradient elution was set from 0 to 1 min, 90% A; 1–12 min, 90–5% A; 12–14 min, 5% A; 14–14.1 min, 5–90% A; 14.1–15 min, 90% A. The samples were dissolved in the initial mobile phase, filtered through a 0.22 μm filter, and injection volume was 5 μL. All determinations were performed in triplicate. MS parameters were as follows: scan range, *m*/*z* 100−1000; ion spray voltage, −4.5 kV in negative mode; source temperature, 500 °C. Nitrogen was used as the ion source gas, curtain gas and collision gas. Gas 1 and gas 2 of the ion source were operated at 50 psi and 55 psi, respectively. SCIEX OS software (version: 1.2.0.4122) was carried out for data processing. Identification of the compounds was performed by comparing their MS/MS spectra with the pure standards with their chemical structure shown in Figure 1B. Quantification was achieved by constructing the calibration curve of each standard at the range of 1 to 100 ng/mL. 

### 2.4. Animals and BBE Treatment

C57BL/6J male mice were obtained from Beijing HFK Biotechnology Co., Ltd., Beijing, China (Aged six to eight weeks, No.110322221103861458), and all animals used in this study were approved by the Ethics Committee of Zhengzhou University (Approval No. ZZUIRB 2021-1201). Thirty mice were divided into three groups at random (*n* = 10) and kept in a pathogen-free environment (12/12 h day/night cycle) with relatively stable temperature (22 ± 2 °C) and humidity (55–65%), and the mice were provided with food (HFK Bioscience CO., Ltd., Beijing, China, 1025) and fresh water ad libitum. After one week acclimatization, the mice were given either water or an BBE solution at doses of 50 mg/kg (BBE50) and 100 mg/kg (BBE100) by gavage daily for eight weeks, and food intake and body weight were recorded weekly. Tissues were collected at the end of the experiments and stored in a refrigerator at −80 °C.

### 2.5. Grip Strength and Treadmill Running Tests

Grip strength and treadmill tests were carried out following the previous studies [11,24]. The grip strength of the forelimbs and limbs was measured with a grip measurement device (SA417). Each mouse was tested three times and the maximum peak force was recorded. For treadmill running test, the mice were put on a treadmill (XR-PT-10B, Shanghai, China) running at a speed of 10 m/min and subjected to run for 10 min per day for three days. In the formal test, the treadmill was run at 10 m/min for the first 10 min, followed by an increase of 1 m every 3 min for low-speed running and 5 m every 2 min for high-speed running.

### 2.6. Body Composition and Magnetic Resonance Imaging (MRI) Analysis

Body composition was measured as previously described using an animal body composition analyzer with an NIMI-SZ01 nuclear magnetic resonance system (Niumag Corporation, Suzhou, China) to quantify lean and fat mass in mice [25]. In brief, prior to the experiment, the mice were fasted for 12 h and anaesthetized, and the whole body of each mouse was scanned in sagittal, axial, and coronal planes. The following settings were used for scanning: TE = 15.24 ms, TR = 400 ms, K space = 192 × 256 µm, FOVRead = 100 mm, FOVPhase = 100 mm, slice thickness = 2.5 mm, and slice counts = 6. Finally, false color was added to the gray image using Niumag NMR Image Processing Software (version 3.0).

### 2.7. Metabolic Cage Monitoring

The metabolic capacity of the mice was examined by using an animal monitoring system (Columbus Instruments, Columbus, OH, USA, CLAMS). Briefly, the mice in each group were randomly selected for adaptation for 24 h in a metabolic cage with free access to water and food, and data on O_2_ consumption (VO_2_), CO_2_ production (VCO_2_), and respiration exchange rate (RER) were automatically collected over the next 48 h.

### 2.8. Enzyme Activities Assay

The activities of lactate dehydrogenase (LDH) and succinate dehydrogenase (SDH) in gastrocnemius tissue were determined according to the method described in the commercial assay kits (Jiancheng Bioengineering Institute, Nanjing, China).

### 2.9. Quantitative Real-Time PCR

Total mRNA was extracted from mice gastrocnemius and soleus muscles using Trizol reagent (Invitrogen, Waltham, MA, USA). The RNA concentration was detected with the NanoDrop2000 (Thermo, Waltham, MA, USA), and complementary DNA synthesis was achieved using the Takara Reverse Transcription Kit (Takara, Otsu, Japan). Primer sequences are shown in Table S1. Real-time PCR was carried out in 96-well plates using the Real-Time System (Bio-Rad, Hercules, CA, USA, CFX96TM) and relative mRNA expression levels were quantified by using the 2^−ΔΔCT^ method. 

### 2.10. Western Blotting

Tissue samples were treated with radioimmunoprecipitation assay (RIPA) buffer containing inhibitors (protease, phosphatase) and centrifuged at 11,500× *g* at 4 °C for 15 min. The concentration of these protein samples was determined by the bicinchoninic acid protein assay kit (Beyotime, Shanghai, China). Denatured protein samples were isolated by SDS-PAGE and transferred onto polyvinylidene difluoride membranes. The membranes were blocked with 10% fat-free milk for 2 h and then placed in primary antibodies specific for MyHC-I (Proteintech, Wuhan, China, 22280-1-AP), MyHC-IIA (Abcam, Cambridge, UK, ab124937), MyHC-IIB (Proteintech, Wuhan, China, 20140-1-AP), Akt (Cell Signaling Technology, 4685S), P-Akt (ser473) (Proteintech, Wuhan, China, 66444-1-Ig), FoxO1 (Proteintech, Wuhan, China, 18592-1-AP), P-FoxO1 (ser256) (Signalway Antibody, Greenbelt, MD, USA, 12198), and GAPDH (Proteintech, Wuhan, China, 60004-1-Ig) overnight at 4 °C and finally incubated in secondary antibodies (Proteintech, Wuhan, China, SA00001-1 or SA00001-2) for 1 h at room temperature. Protein signal was visualized by the SmartChemiTM 610 plus (SinSage Technology, Beijing, China).

### 2.11. Immunofluorescence Staining

For IF staining, the wax blocks were cut into 4 μm on the HistoCore BIOCUT (Leica Biosystems, Shanghai, China) and baked in an oven at 60 °C for 3 h. Antigen retrieval was performed using a sodium citrate antigen retrieval solution (Solarbio, Beijing, China, C1032). Muscle sections were blocked with 3% BSA for 40 min at room temperature and incubated with primary antibodies specific for MyHC-I (Proteintech, Wuhan, China, 22280-1-AP), Fast Myosin Skeletal Heavy Chain (Abcam; ab51263), and Laminin (Abcam, ab11575) overnight at 4 °C, followed by IgG H&L (FITC) (Abcam, ab6717), IgG H&L (Cy3) (Abcam, ab97035) secondary antibodies for 1 h at room temperature. Fluorescence images were collected by a confocal laser microscope (Zeiss, Jena, Germany, LSM880) and processed by Image J software or Zen 2.6 lite.

### 2.12. Molecular Docking

The structures of myricitrin (CID: 5281673), hyperoside (CID: 5281643), myricetin (CID: 5281672), cyanidin-3-O-glucoside (CID: 441667), quercitrin (CID: 5280459), quercetin (CID: 5280343), and kaempferol (CID: 5280863) were acquired from the PubChem database. The structure of Akt (PDB: 3o96) was acquired from the Protein Data Bank database. PyMOL software (version: 2.6.0) was used to remove the water, ligands, and redundant sequences for a stable Akt structure. Molecular docking was carried out by using AutoDock Vina (version 1.1.2). The analysis and visualization of docking results were conducted by using PyMOL software (version: 2.6.0). The best conformations with the highest affinity were selected as the final docking results. 

### 2.13. Statistical Analysis

All data were analyzed by SPSS 21.0 software and were expressed as mean ± SD. Significance was determined by Student’s *t*-test and one-way ANOVA. Statistical significance levels were defined as * *p* < 0.05, ** *p* < 0.01.

## 3. Results

### 3.1. Quantitative Analysis of Flavonoids in “Biqi” Bayberry Extract

The identification and quantification of main flavonoids in “Biqi” bayberry extract was performed by triple quadrupole mass spectrometry, according to mass spectrometry data under negative ESI mode comparing with an authentic standard. The compounds of bayberry extracts eluted with 20% ethanol were presented in Figure 1C. In the extracts, myricitrin was the predominant flavonoside with the amount of 11.07 mg/g, which accounted for 34.6% of the total flavonoids. In another report, myricitrin was also the dominant flavonoid glycoside in the extract of bayberry cultivar “Shuijing” [18]. As the aglycone of myricitrin, myricetin accounted for 19.7% of the detected flavonoids. Myricetin content in our study was much higher than that previously reported by Zhang et al. [26] which may be owing to the fact that we enriched the 20% ethanol eluted fraction of the flavonoids. Quercetin and its derivatives (hyperoside and quercitrin) were proved to be the secondary parts of the bayberry extract, where hyperoside was the predominant ingredient. The results were slightly different from that reported by Liu et al. [18] who found that quercitrin was the major quercetin derivative. The primary reasons could be varietal differences between two cultivars; differences in extraction and determination methods may also contribute to the observed variations. C3G and kaempferol were relatively low compounds in the extract, and C3G has been reported in numerous literatures to be the primary anthocyanin present in bayberry and responsible for the characteristic red color of fruits [27,28]. 

### 3.2. Effects of BBE on the Mass and Function of Skeletal Muscle in Mice

**Figure 2:**
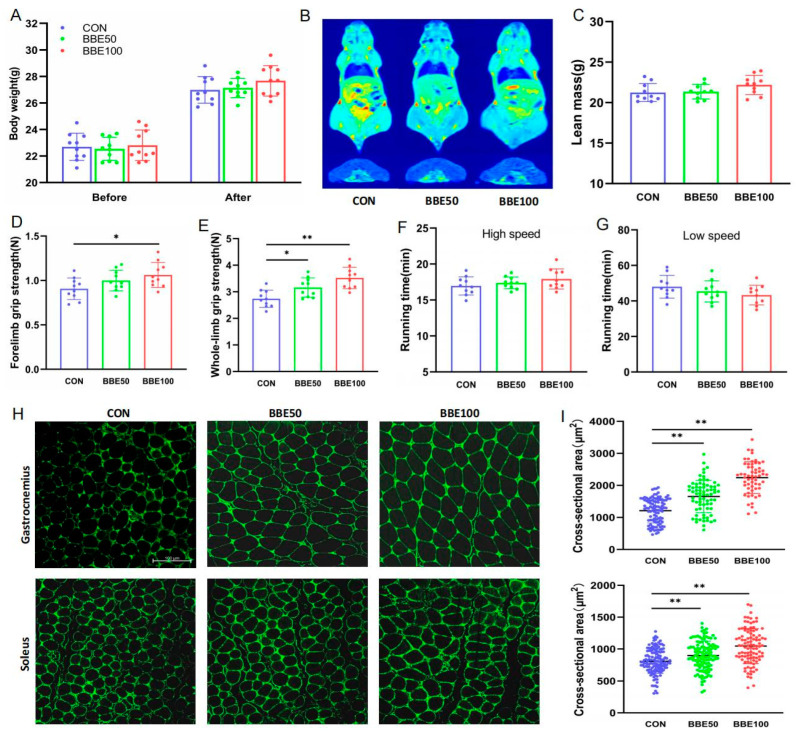
BBE increases the grip strength of skeletal muscle in mice. (**A**) Body weights of control and BBE groups mice (*n* = 10). (**B**) A representative of magnetic resonance imaging. (**C**) Lean mass (*n* = 10). (**D**) Forelimb grip strength (*n* = 10). (**E**) Whole-limb grip strength (*n* = 10). (**F**) High-speed running time (*n* = 10). (**G**) Low-speed running time (*n* = 10). (**H**) Laminin IF staining of gastrocnemius and soleus muscles (Scale bars, 100 μm). (**I**) Frequency distribution of the cross-sectional myofiber area. Results are presented as mean ± SD, (*) *p* < 0.05, (**) *p* < 0.01.

To identify the effects of BBE on skeletal muscle, the mice were treated with 0, 50 mg/kg, or 100 mg/kg BBE for eight weeks. Body weights of the three groups of mice stayed balanced before BBE intervention, while they had a slight increase at the end of the intervention with the increase of the dose (*p* > 0.05) (Figure 2A). There were slightly increasing but no significant trends in lean mass, average daily food intake, gastrocnemius index, and soleus index (*p* > 0.05) (Figure 2C, Figure S1B–D), in line with the skeletal muscle distributions of the whole body (Figure 2B). Meanwhile, we found that BBE supplementation had no effects on fat mass (*p* > 0.05) (Figure S1A). Furthermore, we examined the exercise capacity in mice. As shown in Figure 2D,E, 100 mg/kg BBE supplementation enhanced the forelimb grip strength (*p* < 0.05), and the whole-limb grip strength was consolidated along with the increasing dose of BBE (*p* < 0.05 for BBE50 and *p* < 0.01 for BBE100). Although there was no noticeable difference, there was a tiny variation in high-speed and low-speed running time as the dose escalated (*p* > 0.05) (Figure 2F,G). Immunofluorescence staining further showed that the mean CSA (cross-sectional area) of individual myofibers of gastrocnemius as well as soleus muscles in the BBE50 group and the BBE100 group climbed significantly compared to the control group (*p* < 0.01 for BBE50 and BBE100 in gastrocnemius and soleus muscles) (Figure 2H,I). Accordingly, BBE supplementation ameliorates grip strength capacity and changes muscle fiber size in mice.

### 3.3. BBE Leads to the Transformation of Energy Metabolism Substrate in Mice

**Figure 3:**
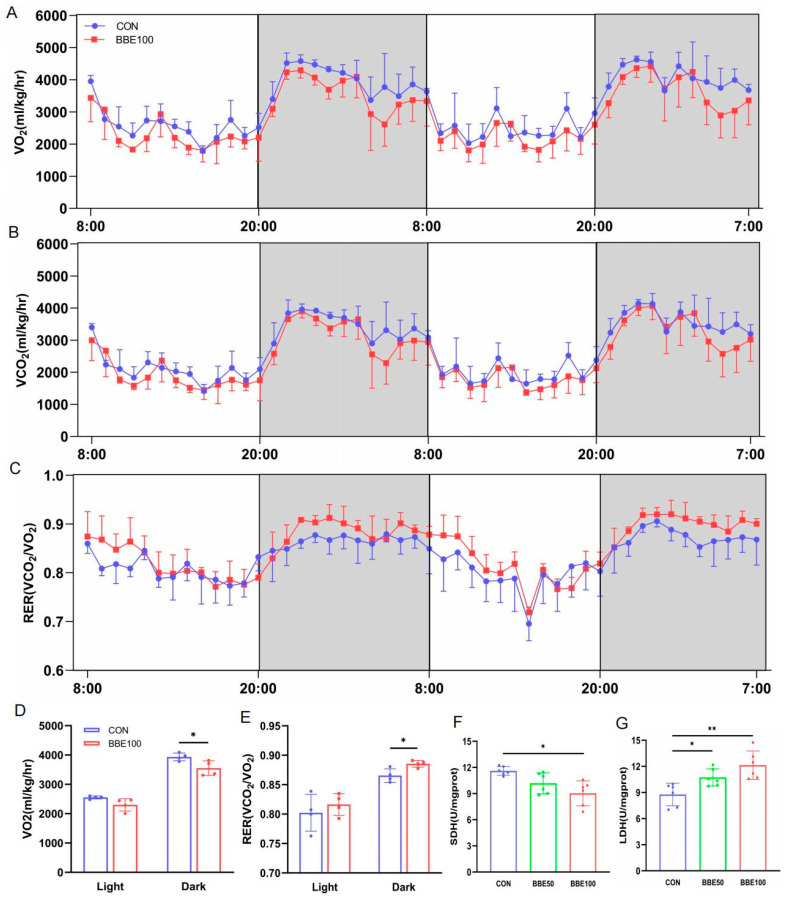
BBE improves glycolytic metabolism capacity in mice. (**A**,**D**) Oxygen consumption of control and BBE100 group mice in 48 h (*n* = 4). (**B**) CO_2_ production (VCO_2_) in 48 h (*n* = 4). (**C**,**E**) Respiratory exchange ratio (RER) in 48 h (*n* = 4). (**F**) SDH activity of gastrocnemius muscles in each group of mice (*n* = 6). (**G**) LDH activity of gastrocnemius muscles in each group of mice (*n* = 6). Results are presented as mean ± SD, (*) *p* < 0.05, (**) *p* < 0.01.

To further investigate the impact of BBE on O_2_ consumption (VO_2_), CO_2_ production (VCO_2_), and respiration exchange rate (RER), we performed metabolic cage studies. BBE100-treated mice, compared with the control group, had an effective augment in RER (VCO_2_/VO_2_) and decrease in VO_2_ during the dark cycle (*p* < 0.05 in RER and VO_2_ for BBE100), which means a shift from fatty acid to glucose as the main energy metabolism substrate (Figure 3A–E). Intriguingly, the BBE100 group exhibited lower SDH activity than the control group (*p* < 0.05), while the activity of LDH increased both in the BBE50-treated and BBE100-treated groups relative to the control (*p* < 0.05 for BBE50 and *p* < 0.01 for BBE100) (Figure 3F,G). These results demonstrate that BBE may promote the switch of aerobic oxidation to glycolytic metabolism in the whole bodies of mice.

### 3.4. BBE Induces the Switch of Slow-to-Fast Fiber Types in the Gastrocnemius of Mice

**Figure 4:**
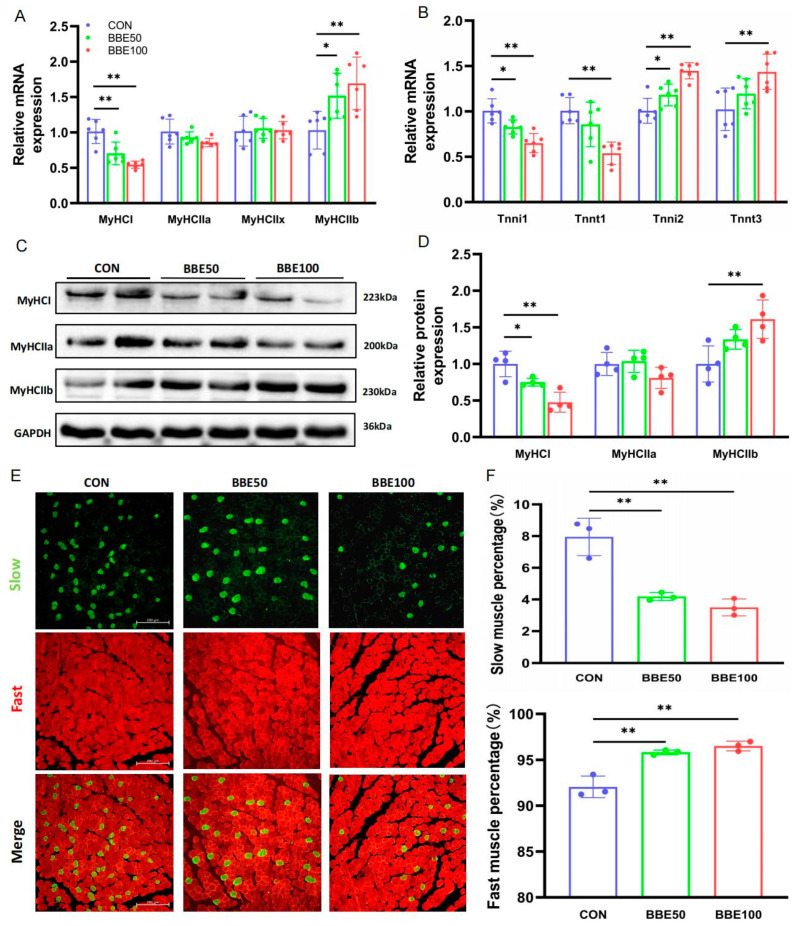
BBE regulates skeletal muscle fiber composition in gastrocnemius muscles of mice. (**A**,**B**) mRNA expression of MyHCI, MyHCIIa, MyHCIIx, MyHCIIb, Tnnt1, Tnni1, Tnnt3, and Tnni2 in gastrocnemius muscles of each group of mice (*n* = 6). (**C**,**D**) Protein expression and quantification of MyHCI, MyHCIIa, and MyHCIIb in gastrocnemius muscles of each group of mice (*n* = 4). (**E**,**F**) Slow and fast muscle fibers IF staining and quantification analysis (*n* = 3, Scale bars, 200 μm). Results are presented as mean ± SD, (*) *p* < 0.05, (**) *p* < 0.01.

In order to explain the conversion above, we analyzed the genes related to slow and fast fibers using qPCR; Western blot and immunofluorescence staining in gastrocnemius were also carried out. As the dose of BBE increased, the mRNA expression of fast fiber-specifics such as MyHCIIb, Tnni2, and Tnnt3 were dramatically upregulated (*p* < 0.05 in MyHCIIb and Tnni2 for BBE50; *p* < 0.01 in MyHCIIb, Tnni2, and Tnnt3 for BBE100), whereas the relative mRNA levels of MyHCI, Tnni1, and Tnnt1 were downregulated (*p* < 0.05 in Tnni1 and *p* < 0.01 in MyHCI for BBE50; *p* < 0.01 in MyHCI, Tnni1 and Tnnt1 for BBE100) (Figure 4A,B). Consistent with the results of qPCR, a reduction in the protein expression levels of MyHCI was observed in BBE-treated mice in a dose-dependent manner (*p* < 0.05 for BBE50 and *p* < 0.01 for BBE100), while the upregulation of MyHCIIb was consistently detected in both treated groups (*p* < 0.01 for BBE100) (Figure 4C,D). Moreover, an immunofluorescence staining assay revealed a decreasing trend in the proportion of slow myofibers and an increase in fast myofibers in mice treated with BBE (*p* < 0.01 for BBE50 and BBE100 in slow and fast myofibers) (Figure 4E,F). All these findings verify that BBE has a positive effect in increasing fast-twitch myofibers proportion in the gastrocnemius of mice. 

### 3.5. BBE Regulates the Switch of Slow-to-Fast Fiber Types in the Soleus of Mice

**Figure 5:**
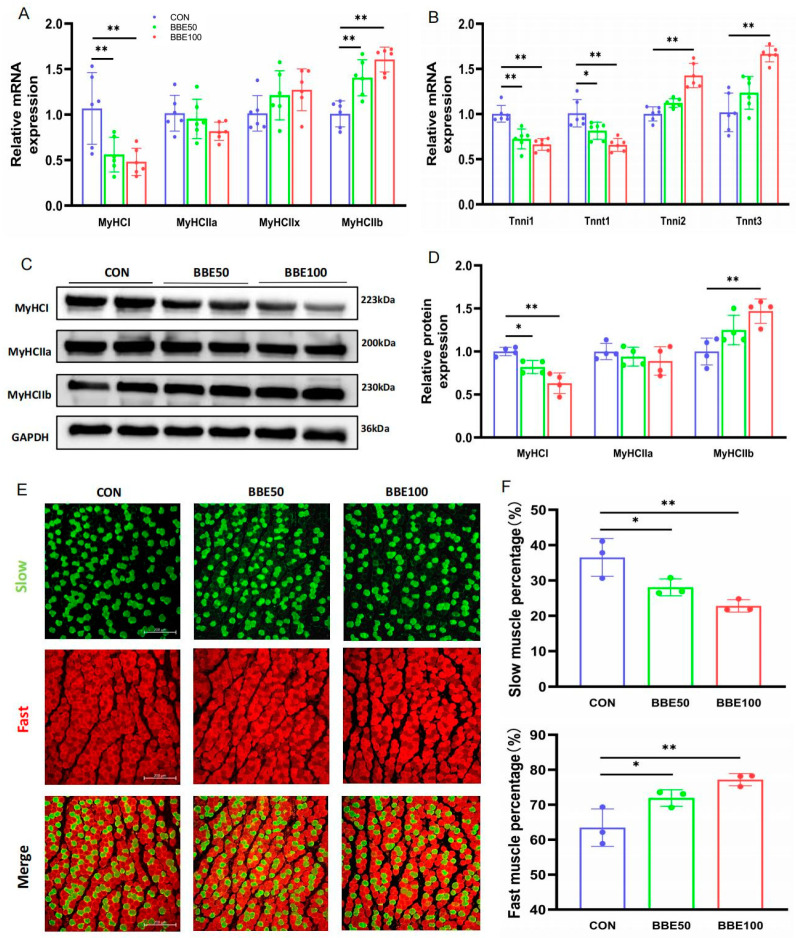
BBE regulates skeletal muscle fiber composition in soleus muscles of mice. (**A**,**B**) mRNA expression of MyHCI, MyHCIIa, MyHCIIx, MyHCIIb, Tnnt1, Tnni1, Tnnt3, and Tnni2 in soleus muscle of each group mice (*n* = 6). (**C**,**D**) Protein expression and quantification of MyHCI, MyHCIIa, and MyHCIIb in soleus muscles of each group of mice (*n* = 4). (**E**,**F**) Slow and fast muscle fibers IF staining and quantification analysis (*n* = 3, Scale bars, 200 μm). Results are presented as mean ± SD, (*) *p* < 0.05, (**) *p* < 0.01.

Likewise, we conducted qPCR, Western blot, and immunofluorescence staining in the soleus and obtained the parallel results that were observed in the gastrocnemius. BBE50 and BBE100 suppressed slow fibers-associated gene expression (both at mRNA and protein level), while concurrently elevating the expression levels of those pertinent to fast fibers (Figure 5A–D). Similarly, the phenotype of myofibers, shown as the percentage of slow or fast myofibers, also changed accordingly (Figure 5E,F). Therefore, we found that BBE has the ability to remodel the skeletal muscle myofibers from slow to fast types in mice. 

### 3.6. BBE Activates the Akt-FoxO1 Pathway in the Gastrocnemius and Soleus of Mice

**Figure 6:**
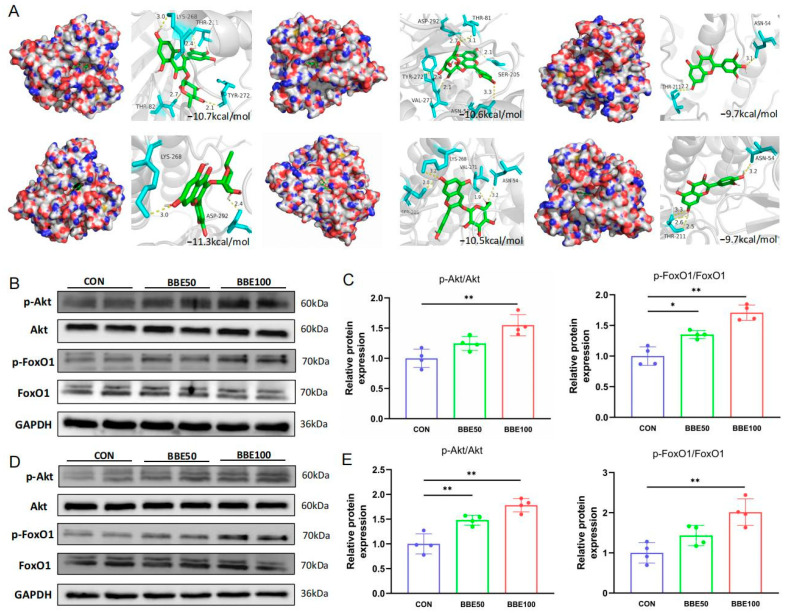
BBE activates the Akt/FoxO1 pathway in skeletal muscles of mice. (**A**) The binding modes of myricitrin, hyperoside, myricetin, quercitrin, cyanidin-3-O-glucoside, and quercetin in the Akt protein. (**B**,**C**) Protein expression and quantification of p-Akt/Akt and p-FoxO1/FoxO1 in the gastrocnemius muscles of each group mice (*n* = 4). (**D**,**E**) Protein expression and quantification of p-Akt/Akt and p-FoxO1/FoxO1 in soleus muscles of each group mice (*n* = 4). Results are presented as mean ± SD, (*) *p* < 0.05, (**) *p* < 0.01.

To investigate whether BBE regulates fast-twitch myofibers switching by activating the Akt-FoxO1 pathway, the binding energy of docking between the compounds (1–7) and Akt was analyzed and the expressions of Akt and FoxO1 were detected. As shown in Figure 6A and Figure S2, these major flavonoids in the bayberry extract could act on Akt with low docking scores ranging from −9.4 to −11.3 kcal/mol. Myricitrin, the most abundant flavonoid in BBE, had superior binding energy with Akt (−10.7 kcal/mol) and displayed strong binding interaction via creating four hydrogen binds with LYS268, THR211, THR82, and TYR272. As expected, compared to the control group mice, BBE supplementation significantly increased p-Akt/Akt (*p* < 0.01 for BBE100) and p-FoxO1/FoxO1 expressions (*p* < 0.05 for BBE50 and *p* < 0.01 for BBE100) in the gastrocnemius muscles of mice (Figure 6B,C). Consistent with this finding, BBE also activated the Akt-FoxO1 pathway in the soleus muscles of mice (Figure 6D,E). Overall, these results suggested that BBE promoted skeletal fast-twitch myofibers switching though activating the Akt-FoxO1 pathway. 

## 4. Discussion

Fast-twitch myofibers, characterized by a greater abundance of glycolytic metabolic enzymes, are known to exert positive effects on improving energy metabolism and maintaining glucose homeostasis [29,30]. Skeletal muscles display a remarkable plasticity in converting between slow-twitch myofibers and fast-twitch types, which can be modulated by certain bioactive compounds derived from plants. Bayberry fruits, containing a high amount of flavonoids such as myricitrin, myricetin, and hyperoside, etc., have been reported to exert antidiabetic effects both in vitro and in vivo [18,19]. However, the effect of bayberry fruits on fast-twitch myofibers expression and myofibers composition has not been investigated so far. In current study, our results showed that BBE supplementation increased the grip strength, VCO_2_/VO_2_ ratio, proportion of large myofibers, and the activity of LDH in mice. Furthermore, the supplementation also upregulated the expression of MyHCIIb (fast) and increased the percentage of MyHCIIb in skeletal muscle. In contrast, BBE downregulated the expression of MyHCI (slow) and reduced the O_2_ consumption, the activity of SDH, and the proportion of slow-twitch myofibers. Molecular analyses showed that BBE could activate the Akt/FoxO1 signaling pathway in the skeletal muscles of mice. These results proved that BBE supplementation can regulate myofibers switching from slow- to fast-twitch types through activating the Akt/FoxO1 pathway.

Fast-twitch myofibers have a large myofiber size, strong explosive strength, and high-level glycolytic metabolism. Studies have proved that increasing fast-twitch myofibers proportion or inducing fast-twitch myofibers switching is associated with the improvement of glucose homeostasis, alleviation of metabolic disease, and prevention of age-related muscle disease [23,31]. Recently, a number of studies showed that certain compounds from fruits, vegetables, and herb resources could exert positive effects on the composition of skeletal muscle fibers and can increase the proportion of fast-twitch myofibers. Wang et al. reported that EGCG reduces the proportion of slow-twitch myofibers and improves glucose metabolic enzyme activity [10]. Lauric acid, as stated by Wang et al., activates MyHCIIb protein expression and increases the ratio of fast-twitch myofibers [13]. Moreover, Li et al. found that geniposide promotes slow-to-fast myofibers type switching and accelerates fast-twitch myofibers formation [5]. In this study, we found that BBE supplementation could markedly increase the activity of glycolytic enzyme LDH, which is recognized as a key indicator of anaerobic glycolytic degree. The extract was also found to activate the fast-twitch related genes expression (MyHCIIb, Tnnt3, and Tnni2), and upregulate MyHCIIb protein expression. Furthermore, BBE can significantly decrease oxidative enzyme SDH activity, reduce slow-twitch related genes expression (MyHCI, Tnnt1, Tnni1), and downregulate MyHCI protein expression. In keeping with these results, the forelimb grip strength, whole-limb grip strength, and muscle fiber size all had a significant increase, and the lean mass had a slight improvement in BBE-group mice, proving that the increased proportion of fast-twitch myofibers may benefit to enhance the grip strength performance. Moreover, consistent with these findings, the oxygen consumption was markedly decreased and RER was greatly increased, indicating a high glycolytic metabolism level in BBE-group mice. Consistent with our findings, previous studies showed that inducing fast-twitch myofibers switching by transgenic models or lentivirus treatment significantly improved the muscle performance with an increase in whole-limb grip strength and regulated the glycolytic metabolism with a decrease in O_2_ consumption and an increase in RER [5,22]. The above results demonstrated that BBE could increase fast-twitch myofibers proportion and promote myofibers type reprogramming.

Forkhead box O1 (FoxO1) plays an indispensable role in controlling myogenic differentiation and maintaining skeletal muscle mass [32]. FoxO1 could regulate Hes1 activity to inhibit myoblast differentiation, which has been viewed as an important target for increasing fast-twitch myofibers formation [5,23,32]. It has been shown that FoxO1 knockout in skeletal muscles of mice displayed a significantly increase of fast-twitch myofibers proportion, and FoxO1 inhibited by pharmacological stimuli also significantly induced myofibers switching from slow-twitch to fast-twitch, both in vivo and in vitro [5,32]. Akt, an important upstream regulator of FoxO1, could inhibit FoxO1 dependent transcription of target genes by phosphorylating the FoxO1 to control its nuclear exclusion and inactivity [33,34]. Although the molecular regulation mechanisms of fast-twitch muscle fiber are not fully understood, the Akt-FoxO1 pathway has been considered as a vital signaling axis in controlling fast-twitch myofibers proportion and regulating myofiber remodeling. In this study, we found that BBE supplementation activated p-Akt/Akt and p-FoxO1/FoxO1 expression both in the gastrocnemius and soleus muscles of mice, indicating that BBE could activate mice’s Akt-FoxO1 pathways. Hence, we inferred that the positive effects of BBE on promoting myofiber remodeling and fast-twitch myofibers switching may associate with the Akt-FoxO1 pathway activating.

Bayberry is a rich source of natural flavonoids that has been reported to improve skeletal muscle and regulate the Akt pathway. In the present study, we identified the major flavonoid compositions in the bayberry extract, which were myricitrin, myricetin, hyperoside, quercetin, quercitrin, kaempferol, and cyanidin-3-O-glucoside. This result is similar to a recent report about the major bioactive substances of bayberry extract [18]. The results of molecular docking further suggested that these major flavonoids from bayberry extract act on Akt with a low affinity (−10.7, −9.7, −10.6, −9.7, −11.3, −9.4, and −10.5 kcal/mol, respectively), indicating that BBE could affect Akt expression. Myricitrin, the most abundant flavonoid in BBE, has been reported to activate Akt signaling for improving cardiomyocytes apoptosis [35]. Myricetin was also reported to regulate Akt signaling for improving skeletal muscle function in fructose-fed rats [36]. Hyperoside was found to affect Akt signaling for exerting effects in vivo and in vitro [37,38]. The positive effect of BBE on the Akt-FoxO1 pathway may be attributed to the presences of myricitrin, myricetin, and hyperoside. Still, further study is needed to investigate the specific mechanism of BBE on myofibers type remodeling.

## 5. Conclusions

In summary, this research identified and quantified the main polyphenolic compounds in “Biqi” bayberry, and investigated the effect and potential mechanism of BBE on myofibers composition. The results suggested that “Biqi” bayberry is abundant in flavonoids such as myricitrin, myricetin, and hyperoside, and BBE regulates skeletal muscle fiber type reprogramming by promoting fast myofiber formation via the Akt/FoxO1 pathway in mice. This study may provide new evidence of bayberry as a functional food ingredient for improving skeletal muscle fitness, and may also provide potential strategy to counteracting diseases caused by fast-twitch myofibers deficiency.

## Data Availability

The data presented in this study are available on request from the corresponding author.

## Supplementary Materials

The following supporting information can be downloaded at: https://www.mdpi.com/article/10.3390/foods12132471/s1, Figure S1: (A) Fat mass of control and BBE groups mice. (B) Average daily food intake of each group of mice. (C) Gastrocnemius index. (D) Soleus index, Figure S2: The binding mode of kaempferol in the Akt protein, Table S1: Primer sequences used in this study.
