# Supplementary material for: “Biqi” Bayberry Extract Promotes Skeletal Muscle Fiber Type Remodeling by Increasing Fast Myofiber Formation via the Akt/FoxO1 Pathway in Mice
Source: Foods. 2023 Jun 23;12(13):2471. doi: 10.3390/foods12132471 (PMC10340394; doi:10.3390/foods12132471)

Table S1. Primer sequences used in this study

Genes	Forward	Reverse
MyHC I	5'-cag aac acc agc ctc atc aac cag-3'	5'-ttc tcc tct gcg ttc cta cac tcc-3'
MyHC IIa	5'-acc gac ttc acc aga ggc atc c-3'	5'-gca cca gcc agc gga aca tc-3'
MyHC IIx	5'-aga cgg aga gga gca gga aga ttg-3'	5'-ttg gtg ttg atg agg ctg gtg ttc-3'
MyHC IIb	5'-cag aca gag agg agc agg aga gtg-3'	5'-ttg gtg ttg atg agg ctg gtg ttc-3'
TNNT1	5'-agg agg agc agg cag aag atg ag-3'	5'-ggg aat caa agg agg cac cac ag-3'
TNNT3	5'-gcg gga gga gga cga gga ag-3'	5'-gtg gct gtc aat gag ggc ttg g-3'
TNNI1	5'-caa gat cac tgc ctc ccg taa act c-3'	5'-tcg ctc ctc gtg ttc ctg ctc-3'
TNNI2	5'-gta tgt ctg ccg acg cca tgc-3'	5'-ccg ttc ctt ctc agt gtc ttc ctt c-3'
36B4	5'-gct tcg tgt tca cca agg agg ac-3'	5'-gtt ctg agc tgg cac agt gac c-3'

Figure S1. (A) Fat mass of control and BBE groups mice. (B) Average daily food intake of each group mice. (C) Gastrocnemius index. (D) Soleus index. Results are presented as mean ± SD, n=10, (*) $p < 0.05$, (**) $p < 0.01$.

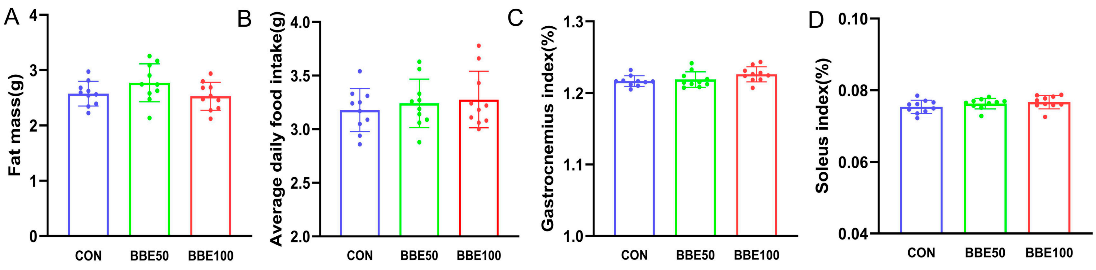

Figure S2. (A) The binding mode of kaempferol in the Akt protein.

A

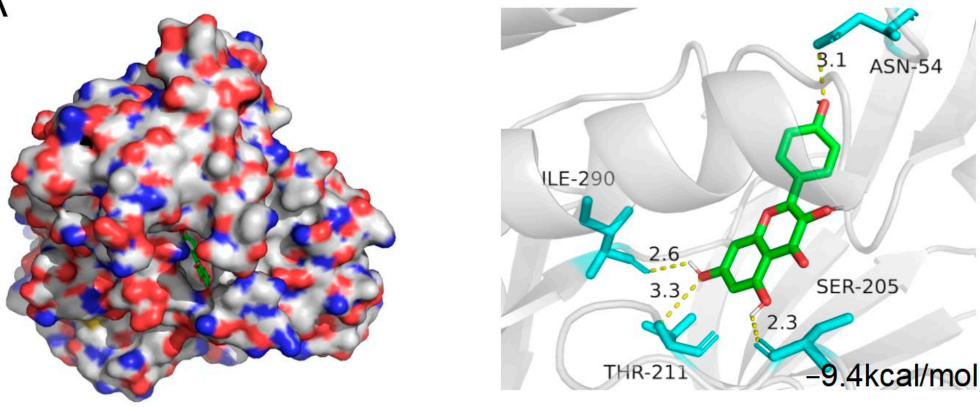

Supplement: Supplementary file 1 [file foods-12-02471-s001.zip › foods-2419348-supplementary.pdf]
